# Air pollution mixture complexity and its effect on PM_2.5_-related mortality: A multicountry time-series study in 264 cities

**DOI:** 10.1097/EE9.0000000000000342

**Published:** 2024-10-30

**Authors:** Pierre Masselot, Haidong Kan, Shailesh K. Kharol, Michelle L. Bell, Francesco Sera, Eric Lavigne, Susanne Breitner, Susana das Neves Pereira da Silva, Richard T. Burnett, Antonio Gasparrini, Jeffrey R. Brook

**Affiliations:** a Environment & Health Modelling (EHM) Lab, Department of Public Health Environments and Society, London School of Hygiene & Tropical Medicine, London, United Kingdom; b Department of Environmental Health, School of Public Health, Fudan University, Shanghai, China; c Environment and Climate Change Canada, Toronto, Ontario, Canada; d AtmoAnalytics Inc., Brampton, Ontario, Canada; e School of the Environment, Yale University, New Haven, Connecticut; f School of Health Policy and Management, College of Health Sciences, Korea University, Seoul, Republic of Korea; g Department of Statistics, Computer Science and Applications “G. Parenti,” University of Florence, Florence, Italy; h School of Epidemiology & Public Health, Faculty of Medicine, University of Ottawa, Ottawa, Canada; i Air Health Science Division, Heatlh Canada, Ottawa, Canada; j IBE-Chair of Epidemiology, LMU Munich, Munich, Germany; k Institute of Epidemiology, Helmholtz Zentrum München – German Research Center for Environmental Health, Neuherberg, Germany; l Department of Epidemiology, Instituto Nacional de Saúde Dr. Ricardo Jorge, Lisbon, Portugal; m Health Canada, Ottawa, Canada; n University of Toronto, Toronto, Ontario, Canada; Department of Epidemiology and Preventive Medicine, School of Public Health and Preventive Medicine, Monash University, Melbourne, Australia, Climate, Air Quality Research Unit, School of Public Health and Preventive Medicine, Monash University, Melbourne, Australia; Center for Climate Change Adaptation, National Institute for Environmental Studies, Tsukuba, Japan; IBE-Chair of Epidemiology, LMU Munich, Munich, Germany, Institute of Epidemiology, Helmholtz Zentrum München – German Research Center for Environmental Health, Neuherberg, Germany; Center for Environmental and Respiratory Health Research (CERH), University of Oulu, Oulu, Finland, Medical Research Center Oulu (MRC Oulu), Oulu University Hospital and University of Oulu, Oulu, Finland; Institute of Atmospheric Physics, Czech Academy of Sciences, Prague, Czech Republic, Faculty of Environmental Sciences, Czech University of Life Sciences Prague, Czech Republic; Institute of Social and Preventive Medicine, University of Bern, Bern, Switzerland, Oeschger Center for Climate Change Research, University of Bern, Bern, Switzerland; Department of Family Medicine and Public Health, University of Tartu, Tartu, Estonia; Estonian Environmental Research Centre, Tallinn, Estonia; Santé Publique France, Department of Environmental and Occupational Health, French National Public Health Agency, Saint Maurice, France; Institute of Epidemiology, Helmholtz Zentrum München – German Research Center for Environmental Health (GmbH), Neuherberg, Germany; Department of Hygiene, Epidemiology and Medical Statistics, National and Kapodistrian University of Athens, Greece, Environmental Research Group, School of Public Health, Imperial College, London, United Kingdom; Department of Hygiene, Epidemiology and Medical Statistics, National and Kapodistrian University of Athens, Greece; Department of Environmental Health, National Institute of Public Health, Cuernavaca, Morelos, Mexico; Department of Environmental Health, National Institute of Public Health, Cuernavaca, Morelos, Mexico; Norwegian institute of Public Health, Oslo, Norway; Department of Environmental Health, Instituto Nacional de Saúde Dr. Ricardo Jorge, Porto, Portugal, EPIUnit - Instituto de Saúde Pública, Universidade do Porto, Porto, Portugal, Laboratório para a Investigação Integrativa e Translacional em Saúde Populacional (ITR), Porto, Portugal; Faculty of Geography, Babes-Bolay University, Cluj-Napoca, Romania; Institute of Environmental Assessment and Water Research (IDAEA), Spanish Council for Scientific Research (CSIC), Barcelona, Spain; Department of Statistics and Computational Research. Universitat de València, València, Spain, CIBERESP, Madrid. Spain; Department of Public Health and Clinical Medicine, Umeå University, Sweden; Swiss Tropical and Public Health Institute, Allschwill, Switzerland, University of Basel, Basel; Department of Environmental Health, Harvard T.H. Chan School of Public Health, Boston, Massachusetts.; Department of Environmental Health, Harvard T.H. Chan School of Public Health, Boston, Massachusetts.

**Keywords:** Fine particulate matter, Mortality, Toxicity, Pollutant mixture, Time series

## Abstract

**Background:**

Fine particulate matter (PM_2.5_) occurs within a mixture of other pollutant gases that interact and impact its composition and toxicity. To characterize the local toxicity of PM_2.5_, it is useful to have an index that accounts for the whole pollutant mix, including gaseous pollutants. We consider a recently proposed pollutant mixture complexity index (PMCI) to evaluate to which extent it relates to PM_2.5_ toxicity.

**Methods:**

The PMCI is constructed as an index spanning seven different pollutants, relative to the PM_2.5_ levels. We consider a standard two-stage analysis using data from 264 cities in the Northern Hemisphere. The first stage estimates the city-specific relative risks between daily PM_2.5_ and all-cause mortality, which are then pooled into a second-stage meta-regression model with which we estimate the effect modification from the PMCI.

**Results:**

We estimate a relative excess risk of 1.0042 (95% confidence interval: 1.0023, 1.0061) for an interquartile range increase (from 1.09 to 1.95) of the PMCI. The PMCI predicts a substantial part of within-country relative risk heterogeneity with much less between-country heterogeneity explained. The Akaike information criterion and Bayesian information criterion of the main model are lower than those of alternative meta-regression models considering the oxidative capacity of PM_2.5_ or its composition.

**Conclusions:**

The PMCI represents an efficient and simple predictor of local PM_2.5_-related mortality, providing evidence that PM_2.5_ toxicity depends on the surrounding gaseous pollutant mix. With the advent of remote sensing for pollutants, the PMCI can provide a useful index to track air quality.

**What this study adds:**

This study assesses to which extent the complexity of the air pollutant mix, including several gaseous pollutants, can explain differential mortality risks of PM_2.5_. It shows that this index can represent an efficient summary of the toxicity of PM_2.5_, especially when comparing cities within the same country.

## Introduction

Fine particulate matter (PM_2.5_) remains one of the most deadly environmental risk factors,^1^ with short-term impacts observed across the globe.^2,3^ Furthermore, several studies have shown that the toxicity of PM_2.5_ varies according to its sources and composition with varying degrees of population vulnerability depending on the location.^4–10^ Indeed, PM_2.5_ occurs within a mixture of pollutant gases and interacts with them through chemical reactions.^11–13^ Emissions of all these pollutants vary across locations,^14^ while there are well-established independent health effects of many pollutant gases such as ozone (O_3_), nitrogen dioxide (NO_2_), and sulfur dioxide (SO_2_).^15–17^ Therefore, more than its composition, the overall mixture in which PM_2.5_ occurs influences its toxicity, and characterizing this mixture can inform about the vulnerability of populations to PM_2.5_.

Different approaches have been proposed to characterize a multipollutant mixture and its effect on health, but results vary given the diversity of methods and availability of pollutant data.^11,18,19^ Harnessing recent advances in remote and satellite-based pollution datasets, Brook and colleagues (Brook JR, Kharol SK, Shephard MW, Sioris CE, McLinden CA.Characterization of air pollution mixtures across the Northern Hemisphere to inform a Multi-Pollutant Index. *Rev*. 2024.) recently proposed a standardized Chronic Air Pollution Index (CAPI) to track exposure to a mixture of seven pollutants (six gases and PM_2.5_) over the Northern Hemisphere. They show a discrepancy between CAPI and PM_2.5_ exposure, confirming that the latter is incomplete in characterizing chronic exposure to air pollution. This work therefore suggests a pollutant mixture complexity index (PMCI) to characterize the mix complexity relative to PM_2.5_ alone.

This contribution aims to assess the effect modification of PM_2.5_-related mortality by the PMCI. The rationale is that, as a measure of the mixture complexity, PMCI could represent PM_2.5_ toxicity in a more comprehensive way than other candidates such as its chemical composition^4,20^ or oxidative capacity.^12,21^ We compare these three different effect modifiers using the extensive Multi-Country Multi-City (MCC) database and using a standard two-stage analysis framework.^22^

## Methods

### Data

#### Multi-Country Multi-City database

The MCC database represents a collection of daily health outcomes and environmental exposures managed by an international network of researchers investigating the association between environmental exposures and human health. We extracted data for cities with both daily mortality and exposure to PM_2.5_ available from the Northern Hemisphere. This results in a sample of 264 cities from 15 countries with periods varying between 1999 and 2018 (Table 1). The outcome was all-cause mortality if available, or nonexternal mortality (International Classification of Diseases 10th Revision: A00-R99) otherwise. The main exposure series is 24-hour average city-level concentrations of PM_2.5_ extracted from nearby monitoring stations. Specific information on the data extraction process from each country can be found in Supplementary Materials A; http://links.lww.com/EE/A304.

**Table 1.** Description of the dataset disaggregated by country

Country	No. cities	Period	Total mortality	Average PM_2.5_ in µg/m^3^ (IQR)	Average PMCI (IQR)
Canada	21	1999–2015	1,915,751	7.26 (6.08–8.48)	1.80 (1.24–2.32)
China	3	2013–2015	244,746	59.22 (47.85–66.59)	0.30 (0.16–0.40)
Estonia	1	2008–2020	12,682	9.38 (9.38–9.38)	1.02 (1.02–1.02)
France	16	2003–2017	976,497	11.92 (9.79–14.93)	1.08 (0.81–1.33)
Germany	11	2004–2020	1,577,189	15.51 (14.56–15.87)	0.89 (0.75–1.06)
Greece	1	2007–2010	114,734	14.60 (14.60–14.60)	0.81 (0.81–0.81)
Mexico	2	2014–2019	78,234	9.35 (8.74–9.96)	1.75 (1.60–1.90)
Norway	1	2000–2018	82,976	7.78 (7.78–7.78)	0.72 (0.72–0.72)
Portugal	1	2004–2018	286,980	9.83 (9.83–9.83)	1.18 (1.18–1.18)
Romania	6	2009–2016	91,090	18.52 (17.07–19.04)	0.34 (0.28–0.45)
Spain	2	2011–2012	8,671	9.35 (9.23–9.47)	1.15 (1.14–1.16)
Sweden	1	2001–2010	82,020	7.05 (7.05–7.05)	1.35 (1.35–1.35)
Switzerland	4	1999–2010	75,518	14.65 (13.85–15.75)	0.65 (0.49–0.74)
United Kingdom	101	2008–2018	2,050,803	10.05 (8.88–11.12)	1.31 (1.07–1.62)
USA, Central	14	1999–2006	890,429	11.27 (10.85–11.80)	1.72 (1.53–1.96)
USA, North-East Central	7	1999–2006	372,918	9.35 (8.40–10.26)	1.99 (1.68–2.10)
USA, North-West Central	1	1999–2006	22,779	9.30 (9.30–9.30)	1.75 (1.75–1.75)
USA, North-East	15	1999–2006	1,282,695	9.06 (8.19–9.80)	2.79 (2.40–3.02)
USA, North-West	6	1999–2006	173,192	7.86 (7.20–7.60)	1.17 (1.02–1.31)
USA, South	12	1999–2006	523,461	9.42 (9.05–10.01)	1.85 (1.74–2.03)
USA, South-East	21	1999–2006	965,765	8.69 (8.25–9.08)	2.23 (1.85–2.57)
USA, South-West	7	1999–2006	235,519	8.44 (7.63–9.33)	1.67 (1.18–1.98)
USA, West	10	1999–2006	1,024,041	11.69 (8.85–14.04)	1.92 (1.61–2.06)
Total	264	1999–2020	13,088,690	10.79 (8.50–11.36)	1.52 (1.09–1.95)

#### Pollutant mixture complexity index

The PMCI is constructed using satellite-derived products for PM_2.5_, NO_2_, SO_2_, O_3_, carbon monoxide (CO), ammonia (NH_3_), and formaldehyde (HCHO). Supplementary Materials B; http://links.lww.com/EE/A304 provide more details on the sources of each pollutant dataset. For each pollutant, annual values were extracted on a common 10 × 10 km grid over North America, Europe, India, and China and averaged over the period 2012–2014. Grid values for the seven pollutants were then used in a principal component analysis and the first component was extracted. This component is scaled to 0–100 scale where 0 represents the least polluted grid point and 100, the most polluted one,^23^ represents the CAPI. It roughly captures 50% of the variability of gaseous pollutants and is especially representative of combustion gases, although all pollutants contribute substantially to the CAPI (see also Supplementary Materials B; http://links.lww.com/EE/A304).

The PMCI is then defined as

$$\begin{array}{l} PMCI=\frac{CAPI-{PM}_{2.5}^{*}}{{PM}_{2.5}^{*}} \end{array}$$ (1)

where ${PM}_{2.5}^{*}$ is the annual PM_2.5_ value rescaled between 0 and 100. The PMCI takes values greater than −1, with PMCI between −1 and 0 indicating CAPI ranks lower than PM_2.5_ and that the location is less polluted than what PM_2.5_ alone suggests, and conversely when PMCI is positive. The PMCI was extracted for each city as the pixel containing the city point location.

#### Other city-level variables

From the NO_2_ and O_3_ values described earlier, we computed their city-specific redox weighted average (O_x_) with the standard formula ($O_{x}=\left(1.07NO_{2}+2.075O_{3}\right)/3.145$).^12^ We also extracted seven PM_2.5_ components from a global reconstruction model^20^: sulfate (SO_4_^2−^), nitrate (NO_3_^−^), ammonium (NH_4_^+^), black carbon, organic carbon, mineral dust, and sea salt. To match a previous analysis,^4^ these components were extracted for each city and averaged for the period 2003–2017.

Finally, we extracted several city-specific characteristics from the Urban Centre Database,^24^ including the Gross Domestic Product per capita for years 2000 and 2015, Normalized Difference Vegetation Index for years 2000 and 2014, and total built-up area for 2000 and 2015. We also computed the average air temperature and temperature range over the whole period from the daily series available within the MCC dataset.

### Statistical methodology

#### Main analysis

The main analysis follows a standard two-stage methodology.^22^ In the first stage, city-specific relative risks (RRs) associated with a 10 µg/m^3^ increase of PM_2.5_ are estimated using a quasi-Poisson regression. Consistently with previous analyses,^2,4^ we included the lag 0-1 moving average of PM_2.5_ as a linear term in the model, along with a day-of-week factor and a natural cubic spline of time with 6 degrees of freedom per year to capture the long-term and seasonal mortality trends. We additionally included a quadratic B-spline of the lag 0-3 moving average of temperature, with knots located at the 10th, 75th, and 90th percentiles of the local temperature distribution.

In the second stage, PM_2.5_ RRs were pooled in a multilevel meta-regression model defined for the city i as:

$$\begin{array}{l} \log\left(RR_{i}\right)\;=\;\log\left(PMCI_{i}\;+\;1\right)+PC1_{i}+PC2_{i}+\xi_{i}+\phi_{c\left(i\right)}\;+\;\epsilon_{i} \end{array}$$ (2)

The log term of PMCI has been selected to acknowledge its lower bound at −1, and because it was the specification minimizing the Akaike information criterion (AIC) among those considered (see Supplementary Materials C; http://links.lww.com/EE/A304). PC1_*i*_ and PC2_*i*_ represent the first two principal components from the Urban Centre Database variables described earlier, temperature average and range, and average PM_2.5_ mass. These terms capture potential confounding by local socioeconomic and environmental characteristics. $\xi_{i}$, $\phi_{c\left(i\right)}$, and $\epsilon_{i}$, respectively, represent city-level and country-level random effects and the residuals of the model. These terms capture differences between cities, such as the different periods covered by data in each country. Because of important differences in climate and pollutant sources across the country, the USA is further subdivided into nine regions, namely Central, North-East Central, North-West Central, North-East, North-West, South, South-East, South-West, and West. From this model, we can then compute the best linear unbiased predictions of log(RR_*i*_).

We report effect modification as the relative excess risk (RER)^25^ for an interquartile range (IQR) increase of the (log) PMCI. The RER is defined as the ratio between predicted RRs at the 75th and 25th percentile of PMCI, with other components of equation 2 set to zero.

#### Model comparison

The main model (2) is compared to a model that directly includes the gaseous pollutants, as well as models containing alternative measures of toxicity of the pollutant mix: (i) a linear term of O_x_, representing a linear interaction with PM_2.5_,^12,13^ and (ii) PM_2.5_ composition integrated through the additive log-ratio transformation.^4,26^ These three models, as well as the main one, are compared to a null model containing no measure of toxicity of the pollutant mix. All the compared models nonetheless contain all other terms (PC1_*i*_, PC2_*i*_, and random effects) shown in (2).

Comparison between models is made through the likelihood ratio test (LRT), the AIC, and the Bayesian information criterion (BIC). Note that we use the corrected version of the AIC for small sample sizes (sometimes referred to as the AIC_c_).^27^

#### Sensitivity analysis

As there is evidence that O_3_ and NO_2_ both influence the effect of PM_2.5_ and have independent effects on mortality,^15,28^ we perform another analysis with these two pollutants added as confounders in a three-pollutant model in the first stage. These two pollutants are added as linear terms of their two-day moving average in the same fashion as the PM_2.5_ term. This sensitivity analysis reduces the number of available cities to 133 due to different data availability between countries.

## Results

### Study area description

We analyzed more than 13 million deaths from 264 cities, with the vast majority coming from Western Europe and North America (Table 1). The average PM_2.5_ concentration was 10.79 µg/m^3^ (IQR: 8.50–11.36) with 55% of locations below 10 µg/m^3^ on average. The highest PM_2.5_ levels in our sample are found in China with an average concentration of 59.22 µg/m^3^ (IQR: 47.85–66.59) followed by Romania (18.52 µg/m^3^, IQR: 17.07–19.04) and Germany (15.51 µg/m^3^, IQR: 14.56–15.87).

The PMCI is positive in all cities of our sample, with an average of 1.52 (IQR: 1.09–1.95), indicating generally complex pollutant mixtures, which is expected since our sample mostly includes large cities. Eastern USA and Canada generally show the highest PMCI values, while China and Romania show values much closer to zero (0.30 and 0.34, respectively).

### Main model

We estimated an RER of 1.0042 (95% confidence interval [CI: 1.0023, 1.0061) associated with an IQR increase of the PMCI (Table 2). This corresponds to predicted PM_2.5_-related mortality RRs of 1.0059 (95% CI: 1.0034, 1.0083) at the 25th PMCI percentile and 1.0101 (95% CI: 1.0072, 1.0129) at the 75th percentile. This is estimated as a strong association by the LRT with a *P* value below 0.0001. By comparison, the null model estimates an average RR of 1.0066 (95% CI: 1.0040, 1.0092) suggesting that the PMCI is associated with a substantial part of the between-location heterogeneity.

**Table 2.** Results of the main and benchmark models, including the relative excess risk (RER) associated with an interquartile range increase of the effect modifier, as well as the *P* value from a likelihood ratio test (LRT), the (corrected) Akaike information criterion (AIC), and Bayesian information criterion (BIC)

Model	RER (95% CI)	LRT *P* value	AIC	BIC
Main		**0.0000**	**−1566.22**	**−1545.09**
PMCI	1.0042 (1.0023, 1.0061)			
Null			−1550.96	−1533.31
Gas mixture		0.0006	−1561.82	−1523.54
NO_2_	0.9994 (0.9983, 1.0005)			
SO_2_	0.9990 (0.9978, 1.0002)			
O_3_	0.9995 (0.9982, 1.0008)			
HCHO	1.0028 (1.0013, 1.0044)			
CO	1.0015 (1.0003, 1.0026)			
NH_3_	0.9999 (0.9997, 1.0001)			
O_x_		0.6883	−1549.03	−1527.90
O_x_	0.9996 (0.9978, 1.0014)			
PM_2.5_ composition		0.0096	−1555.06	−1516.77
SO_4_^2-^	1.0017 (0.9945, 1.0090)			
NH_4_^+^	1.0031 (1.0002, 1.0059)			
NO_3_^-^	0.9971 (0.9955, 0.9987)			
BC	1.0020 (0.9992, 1.0049)			
OC	1.0011 (0.9991, 1.0031)			
SS	0.9977 (0.9913, 1.0042)			
DUST	0.9882 (0.9738, 1.0029)			

Best values for each criterion are indicated in bold.

BC, black carbon; DUST, mineral dust; OC, organic carbon; SS, sea salt.

Figure shows the best linear unbiased predictions from the main model along with the association between PMCI and PM_2.5_-related mortality RR. It suggests that the PMCI captures most of the within-country variability, but that substantial between-country variability remains. This is shown by a more substantial drop in the estimated standard deviation of the city-level random component $\xi_{i}$ (from 0.0027 in the null model to 0.0018) compared to the country-level one (from 0.0046 to 0.0041, see Table S4; http://links.lww.com/EE/A304). Countries such as Canada, Greece, Switzerland, and Eastern USA tend to have high RRs while the United Kingdom, France, Romania, and Western USA have low RRs.

**Figure.**
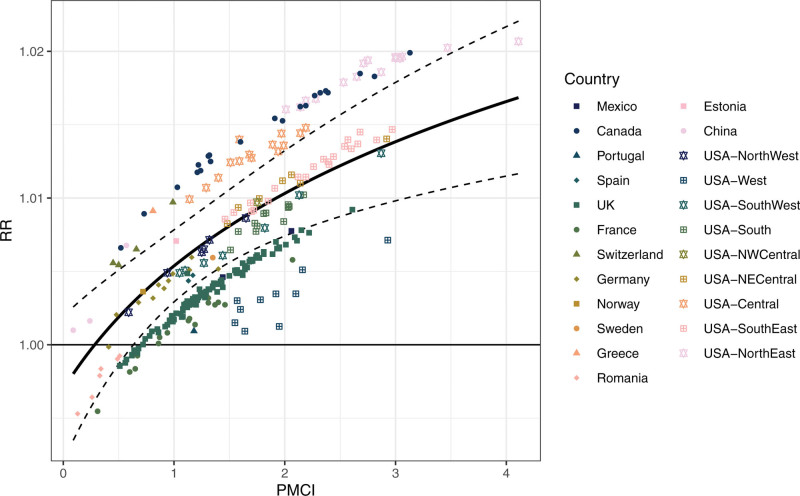
Estimated association between the PM_2.5_ relative risk (RR) and the pollutant mixture complexity index (PMCI). Points represent the best linear unbiased prediction (BLUP) at each city from the main second-stage meta-regression model.

### Model comparison

Table 2 shows the results for the alternative second-stage meta-regression models of PM_2.5_ toxicity. Considering the individual gaseous pollutants suggests evidence of effect modification due to HCHO and CO with RERs of 1.0028 (95% CI: 1.0013, 1.0044) and 1.0015 (95% CI: 1.0003, 1.0026), respectively. In our dataset, O_x_ is not associated with the PM_2.5_-related RR with a RER of 0.9996 (0.9978, 1.0014). In contrast, the LRT suggests that the PM_2.5_ composition model is predictive of PM_2.5_-related RR (*P* value: 0.0096) through associations with ammonium (NH_4_^+^) and nitrates consistently with previous research.^4^ Table S4; http://links.lww.com/EE/A304 also indicates that PM_2.5_ composition reduces mostly the country-level heterogeneity but not the city-level one, in contrast to the PMCI and gas mixture models.

Among all the tested models, the main model (PMCI) has the lowest AIC and BIC (Table 2). It substantially improves upon the null model by showing an AIC difference of around 15 (−1566.55 vs. −1551.19) and shows a difference of 11.16 with the PM_2.5_ composition model, a difference considered important.^29^ It also shows a noticeable difference with the gas mixture model, suggesting that the PMCI provides additional predictive power compared to the gas mixture only. The difference is larger with the BIC as it penalizes more strongly the number of variables in a model.

### Sensitivity analysis

The results obtained by controlling for O_3_ and NO_2_ in the first stage are consistent with the main results, with an estimated RER of 1.0037 (1.0010, 1.0065). There are slightly larger CIs due to the reduced power following the decrease in city availability (see Supplementary Materials F; http://links.lww.com/EE/A304). Interestingly, both the gas mixture and PM composition models now show a lower AIC than the PMCI model (Table S5; http://links.lww.com/EE/A304), while the ranking of the model by BIC remains identical due to the larger penalization of the number of parameters. The O_x_ model still does not provide evidence of effect modification by O_x_ in this dataset (RER: 0.9981, 95% CI: 0.9956, 1.0007).

## Discussion

In this contribution, we evaluated how the PMCI, an index of the relative complexity of the pollutant mix, is associated with the estimated PM_2.5_-related mortality risks. Results indicate that the PMCI predicts a substantial part of the differential risks of PM_2.5_. More specifically, the PMCI was associated with a large part of city-level heterogeneity, while important country and/or regional-level heterogeneity remains. Comparison with other proxies of PM_2.5_ toxicity, as well as the full gas mixture, indicates a higher predictive power of the PMCI on city-specific PM_2.5_ RRs, especially compared to O_x_. These results suggest that the PMCI effectively represents PM_2.5_ toxicity within a simple index.

The CAPI, and by extension the PMCI, shows its highest correlations with HCHO, NO_2,_ and CO (Table S2; http://links.lww.com/EE/A304), the two latter being often associated with combustion and traffic.^11^ Both gases have been strongly associated with mortality^15,30^ and tend to slightly attenuate PM_2.5_ effects when adjusted for.^2,19,31,32^ A synergistic effect of PM_2.5_ and NO_2_ on the cardiovascular system has also been identified by toxicology studies.^33^ This interaction is therefore represented by the effect modification we found for the PMCI. An exploration of effect modification by a single gaseous pollutant indicates the potential effects of CO and HCHO. However, RER and AIC are stronger for the PMCI model than any other in our dataset, confirming that considering pollutants all together is crucial.

Conducting comparisons with alternative toxicity indicators, we found no evidence of effect modification by O_x_, contrary to previous research.^12,13^ The key difference is that previous research assessed interactions with daily variations of O_x_, while we only used an interannual average. This suggests that short-term variations in the oxidative capacity can influence the short-term toxicity of PM_2.5_, but the information provided by its average levels on comparing air quality spatially is limited.

In contrast to O_x_, the results of the effect modification of PM_2.5_ by its composition in our dataset are broadly consistent with previous research.^4,6^ However, we found its effect modification to be slightly less strong than the PMCI’s, suggesting that the seven available components provide an imperfect picture of the toxicity of PM_2.5_. Indeed, the composition is strongly influenced by gases, and considering these gases in addition to PM_2.5_ might provide a fuller characterization of the toxicity. Note that the difference with the PMCI model is less clear-cut when controlling for O_3_ and NO_2_ in the first stage.

While models focusing on effect modification of PM_2.5_ composition or any other chemical air pollutant^9,34^ can inform on the causal pathways of PM_2.5_ toxicity, our study focuses more on the predictive power of PM_2.5_ mortality risks. In addition, the assessed toxicity is at the city level and can be different than the toxicity at the individual level. In this regard, the PMCI (and CAPI) provides little information on causality but summarizes efficiently the PM_2.5_ toxicity through the gas pollutant mixture. It can therefore be a useful ecological indicator of air quality in relation to human health. Computation and tracking of such an indicator is additionally strengthened by the recent progress in remote sensing of various pollutants.^35–37^

The generalizability of the reported results is limited as the dataset is skewed toward North American and European countries, more specifically the USA and the United Kingdom. This is not representative of the existing range of pollutant levels and mix,^38^ as illustrated by the three Chinese cities included in the dataset. Extending this study would be hindered by the limited availability of pollutant and mortality data in countries from the Global South. There are also limitations in the temporal availability of data, resulting in different periods between countries, some of them aligning poorly with the timeframe of the PMCI estimation (Table 1). This aspect is nonetheless controlled for by the country-level random effects. Finally, residual confounding by unmeasured city-level characteristics cannot be excluded given the limited number of variables available in all considered cities.

In conclusion, we show that the PMCI, an indicator of the relative pollutant mixture complexity, represents an efficient predictor of PM_2.5_ risks on mortality. More specifically, the PMCI allowed explaining most of the within-country differentials in risk, while some between-country heterogeneity remains. We additionally show that the PMCI improves upon individual pollutant gases and PM_2.5_ components as an effect modifier. To expand on the usefulness of the PMCI for air quality characterization, future studies should look at other health outcomes. These include cause-specific mortality (e.g., respiratory and cardiovascular), hospital admissions, and subgroups by sex and age.

## Conflicts of interest statement

The authors declare that they have no conflicts of interest with regard to the content of this report.

## ACKNOWLEDGMENTS

The authors like to thank Chris McLinden, Christopher Sioris, and Mark Shephard, who helped with the construction of the CAPI and PMCI indices.

MCC Collaborative Research Network:

Yuming Guo, Department of Epidemiology and Preventive Medicine, School of Public Health and Preventive Medicine, Monash University, Melbourne, Australia, Climate, Air Quality Research Unit, School of Public Health and Preventive Medicine, Monash University, Melbourne, Australia; Yasushi Honda, Center for Climate Change Adaptation, National Institute for Environmental Studies, Tsukuba, Japan; Veronika Huber, IBE-Chair of Epidemiology, LMU Munich, Munich, Germany, Institute of Epidemiology, Helmholtz Zentrum München – German Research Center for Environmental Health, Neuherberg, Germany; Jouni J. K. Jaakkola, Center for Environmental and Respiratory Health Research (CERH), University of Oulu, Oulu, Finland, Medical Research Center Oulu (MRC Oulu), Oulu University Hospital and University of Oulu, Oulu, Finland; Aleš Urban, Institute of Atmospheric Physics, Czech Academy of Sciences, Prague, Czech Republic, Faculty of Environmental Sciences, Czech University of Life Sciences Prague, Czech Republic; Ana Maria Vicedo-Cabrera, Institute of Social and Preventive Medicine, University of Bern, Bern, Switzerland, Oeschger Center for Climate Change Research, University of Bern, Bern, Switzerland; Hans Orru, Department of Family Medicine and Public Health, University of Tartu, Tartu, Estonia; Marek Maasikmets, Estonian Environmental Research Centre, Tallinn, Estonia; Mathilde Pascal, Santé Publique France, Department of Environmental and Occupational Health, French National Public Health Agency, Saint Maurice, France; Alexandra Schneider, Institute of Epidemiology, Helmholtz Zentrum München – German Research Center for Environmental Health (GmbH), Neuherberg, Germany; Klea Katsouyanni, Department of Hygiene, Epidemiology and Medical Statistics, National and Kapodistrian University of Athens, Greece, Environmental Research Group, School of Public Health, Imperial College, London, United Kingdom; Evangelia Samoli, Department of Hygiene, Epidemiology and Medical Statistics, National and Kapodistrian University of Athens, Greece; Magali Hurtado Diaz, Department of Environmental Health, National Institute of Public Health, Cuernavaca, Morelos, Mexico; Eunice Elizabeth Félix Arellano, Department of Environmental Health, National Institute of Public Health, Cuernavaca, Morelos, Mexico; Shilpa Rao, Norwegian institute of Public Health, Oslo, Norway; Joana Madureira, Department of Environmental Health, Instituto Nacional de Saúde Dr. Ricardo Jorge, Porto, Portugal, EPIUnit - Instituto de Saúde Pública, Universidade do Porto, Porto, Portugal, Laboratório para a Investigação Integrativa e Translacional em Saúde Populacional (ITR), Porto, Portugal; Iulian-Horia Holobaca, Faculty of Geography, Babes-Bolay University, Cluj-Napoca, Romania; Aurelio Tobias, Institute of Environmental Assessment and Water Research (IDAEA), Spanish Council for Scientific Research (CSIC), Barcelona, Spain; Carmen Íñiguez, Department of Statistics and Computational Research. Universitat de València, València, Spain, CIBERESP, Madrid. Spain; Bertil Forsberg, Department of Public Health and Clinical Medicine, Umeå University, Sweden; Martina S. Ragettli, Swiss Tropical and Public Health Institute, Allschwill, Switzerland, University of Basel, Basel; Antonella Zanobetti, Joel Schwartz, Department of Environmental Health, Harvard T.H. Chan School of Public Health, Boston, Massachusetts.

## Supplementary Material
