# Supplementary material for: Air pollution mixture complexity and its effect on PM2.5-related mortality: A multicountry time-series study in 264 cities
Source: Environ Epidemiol. 2024 Oct 30;8(6):e342. doi: 10.1097/EE9.0000000000000342 (PMC11527422; doi:10.1097/EE9.0000000000000342)
Supplement: Supplementary file 1 [file ee9-8-e342-s001.docx]

**Supplementary Materials**

# Multi-country multi-city (MCC) data collection

## A.1. Mortality

We obtained mortality data from the Multi-City Multi-Country (MCC) database. The current analysis was limited to cities that have air pollution, temperature, urban characteristics indicator and PMCI data. It includes a total of 264 urban areas in 15 countries/regions (Table 1): Canada (21 cities, 1999-2015), China (3 cities, 2013–2015), Estonia (1 city, 2008–2020), France (16 cities, 2003–2017), Germany (11 cities, 2004-2020), Greece (1 city, 2007–2010), Mexico (2 cities, 2014–2019), Norway (1 city, 2000-2018), Portugal (1 city, 2004–2018), Romania (6 cities, 2009–2016), Spain (2 cities, 2011–2012), Sweden (1 county, 2001–2010), Switzerland (4 cities, 1999–2010), United Kingdom (101 cities, 2008–2018), and United States (93 cities, 1999–2006).

In the present study, mortality is represented by daily counts of either non-external causes (International Classification of Diseases, ICD-9: 0-799; ICD-10: A00-R99) or, where not available, all-cause only. Countries/regions with mortality from non-external causes include: Australia, China and Spain.

## A.2. Exposure

We obtained daily 24-h average concentrations of PM_2.5_ in 264 cities as well as daily 24-h average of NO_2_ concentration and maximum 8-h average of O_3_ in 133 of those cities (details in Table S1). We also collected daily mean temperature for the 264 cities in the analysis. In brief, measurements for air pollutants were obtained from fixed site monitoring networks operated by local authorities or, when available, gridded products (UK). The majority of monitors were located in urban areas, and only those daily measurements reporting above 75% of hourly data were included. On average, there were 4.7 monitors per city (ranging from 1 to 28), and measurements were averaged among all available monitors within one city to represent the exposure levels of the general population.

**Table S1.** Summary of the dataset restricted to locations with daily NO_2_ and O_3_ series available.

| Country | Number of cities | Average NO_2_ in ppbv (IQR) | Average O_3_ in ppbv (IQR) |
| --- | --- | --- | --- |
| Canada | 20 | 3.08 (0.75 - 4.19) | 26.98 (25.14 - 30.58) |
| China | 3 | 35.99 (32.08 - 40.32) | 45.88 (45.40 - 46.41) |
| France | 14 | 5.95 (2.05 - 7.58) | 38.99 (36.98 - 40.35) |
| Germany | 10 | 9.79 (6.94 - 11.24) | 37.36 (36.60 - 38.14) |
| Mexico | 2 | 2.55 (1.76 - 3.34) | 33.66 (30.89 - 36.43) |
| Portugal | 1 | 2.88 (2.88 - 2.88) | 41.19 (41.19 - 41.19) |
| Romania | 4 | 1.47 (1.35 - 1.61) | 41.18 (40.79 - 41.31) |
| Spain | 3 | 1.98 (1.35 - 2.66) | 41.01 (39.58 - 41.99) |
| USA-Central | 10 | 4.98 (3.11 - 6.16) | 29.33 (28.52 - 29.99) |
| USA-NECentral | 5 | 3.25 (1.36 - 3.36) | 31.09 (30.64 - 31.37) |
| USA-NorthEast | 14 | 5.33 (2.50 - 5.07) | 30.66 (29.10 - 32.21) |
| USA-NorthWest | 2 | 5.93 (5.09 - 6.78) | 19.79 (18.14 - 21.45) |
| USA-South | 11 | 1.87 (1.18 - 2.27) | 31.43 (30.08 - 31.68) |
| USA-SouthEast | 17 | 1.99 (0.85 - 2.31) | 26.69 (22.59 - 30.47) |
| USA-SouthWest | 7 | 3.17 (1.23 - 3.91) | 37.40 (36.84 - 40.04) |
| USA-West | 10 | 4.91 (1.73 - 7.03) | 33.59 (29.85 - 38.57) |
| **Total** | **133** | **4.88 (1.41 - 5.67)** | **32.39 (28.51 - 37.50)** |

# The Pollutant Mixture Complexity Index

The Pollutant Mixture Complexity Index is derived from six gaseous pollutants (NO_2_, SO_2_, HCHO, NH_3_, CO, O_3_) and fine particulate matter (PM_2.5_).^1^ It is constructed by first taking the first principal component (PC) of these 7 (standardised) pollutants and scaling it to a 0-100 scale, yielding a Chronic Air Pollution Index (CAPI). The first PC included 50.5% of the seven pollutants variability. The CAPI indicates the overall amount of pollution with more emphasis on combustion gases as indicated in Table S**2**. The PMCI is obtained by comparing CAPI to PM_2.5_ also scaled between 0 and 100 ($PM_{2.5}^{*}$) as

|  | $\frac{CAPI-PM_{2.5}^{*}}{PM_{2.5}^{*}}$ | (1) |
| --- | --- | --- |

When the PMCI is around 0, this means PM_2.5_ summarises the overall air pollution appropriately. When PMCI > 0, the location is more polluted than what PM_2.5_ alone suggests and conversely when PMCI < 0. Note that since both CAPI and PM_2.5-sc_ are scaled between 0 and 100, this means that CAPI is bounded to -1 on the left.

Figure **S1** shows the PMCI and pollutants in each city. It shows for example that the PMCI is low in Chinese and Romanian cities since PM_2.5_ and all other pollutant are equally high. On the other hand, PMCI values are substantially above 1 in Eastern USA since PM_2.5_ tend to be relatively low (just above 10µg/m^3^) while other pollutants such as CO and SO_2_ present relatively high values compared to the other locations.

**Table S****2.** Pollutants included in the Pollutant Mixture Complexity Index (PMCI) with the associated principal component (PC) components.

| Pollutant | Unit | PC coefficient |
| --- | --- | --- |
| Fine particulate matter (**PM_2.5_)** | µg/m^3^ | 0.42 |
| Nitrogen dioxide (**NO_2_**) | ppbv | 0.38 |
| Ozone (**O_3_**) | Ppbv | 0.37 |
| Sulfur dioxide (**SO_2_**) | DU | 0.33 |
| Formaldehyde (**HCHO**) | molecules/cm^2^ | 0.39 |
| Carbon monoxide (**CO**) | ppbv | 0.40 |
| Ammonia (**NH_3_**) | ppbv | 0.33 |


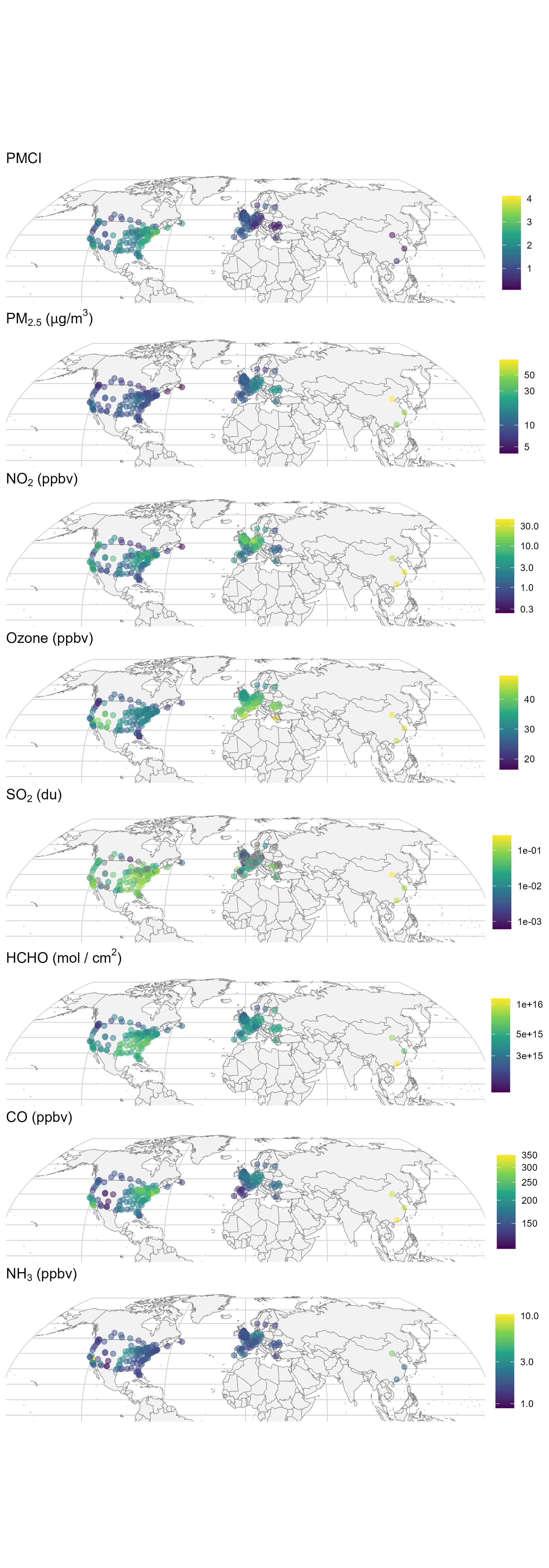


**Figure** **S1.** The Pollutant Mixture Complexity Index (PMCI) and the seven considered pollutants in all cities considered in the analysis. Note that all scale, except for PMCI and Ozone are log_10_ transformed.

# Model Selection

The PMCI is included as a meta-predictor in the second-stage meta-regression model. We considered four different models for the PMCI: i) a linear model, ii) a log-linear model that account for the fact the PMCI is always above -1 (defined as $log(PMCI+1$)), iii) a natural spline with two degrees of freedom, and iv) a natural spline with three degrees of freedom. The fitted association for each model is shown in Figure S2.

The results of model selection are shown in Table S3 and suggests that the log-linear model is the most appropriate. The BIC clearly points towards this model (with a posterior probability of 79%^2^). The AIC is lower than the linear by more than two indicating substantial improvement of the fit.^3^ Note that considering a nonlinear model increases estimated between country heterogeneity while decreasing within country heterogeneity.


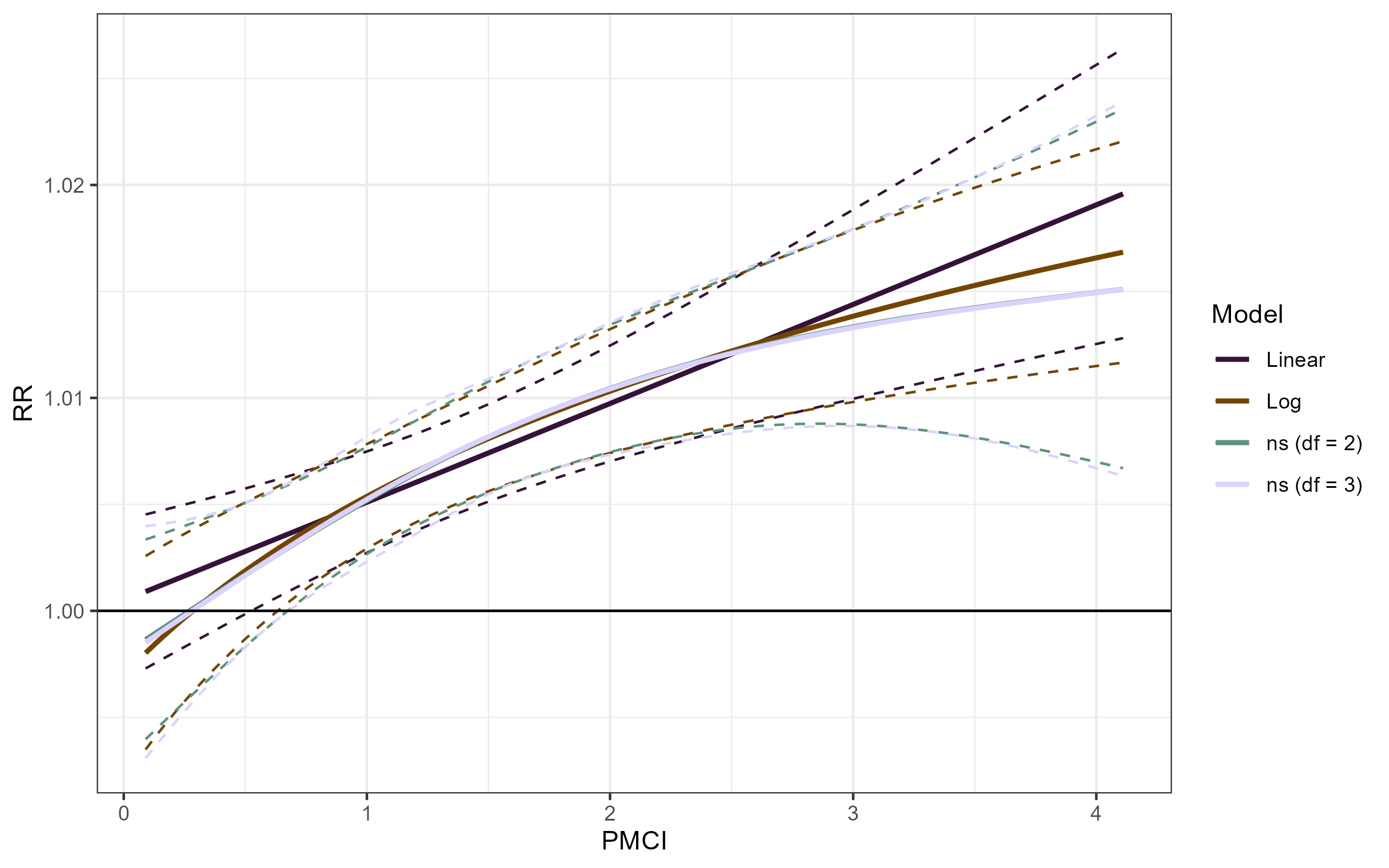


**Figure S****2.** Estimated association between PMCI and PM_2.5_-mortality relative risk (RR) according to each compared model.

**Table S****3.** Model selection for the PMCI including the (corrected) Akaike Information Criterion (AIC), Bayesian Information Criterion (BIC) and estimated random effect standard deviations.

| Model | AIC | BIC | Country Std. Dev. | City Std. Dev. |
| --- | --- | --- | --- | --- |
| Linear | -1563.44 | -1542.32 | 0.0037 | 0.0023 |
| Log | **-1566.22** | **-1545.09** | 0.0041 | 0.0018 |
| ns (df = 2) | -1563.49 | -1538.90 | 0.0041 | 0.0019 |
| ns (df = 3) | -1561.42 | -1533.38 | 0.0041 | 0.0019 |

# Additional results from the main analysis

**Table S****4.** Additional results from the second-stage models. Country and city standard deviations refer to the estimated parameters for the corresponding random effect levels.

| Model | Cochran's Q | I^2^ | Country Std. Dev. | City Std. Dev. |
| --- | --- | --- | --- | --- |
| Main | 372.21 | 30.15 | 0.0041 | 0.0018 |
| Null | 407.29 | 35.92 | 0.0046 | 0.0027 |
| Gas Mixture | 298.05 | 14.44 | 0.0035 | 0.0001 |
| O_x_ | 407.22 | 36.15 | 0.0045 | 0.0027 |
| PM_2.5_ Composition | 305.47 | 16.52 | 0.0021 | 0.0024 |

# Residual Analysis

Figure S3 shows marginal residuals (difference between response and fixed effects) versus several relevant city characteristics. Overall, there is little evidence of additional nonlinearity and an important part of the remaining variation can be captured by the country level random effects. Typically, the PMCI alone seems to slightly underestimate the PM_2.5_-related mortality RR in northeast USA and Canada.


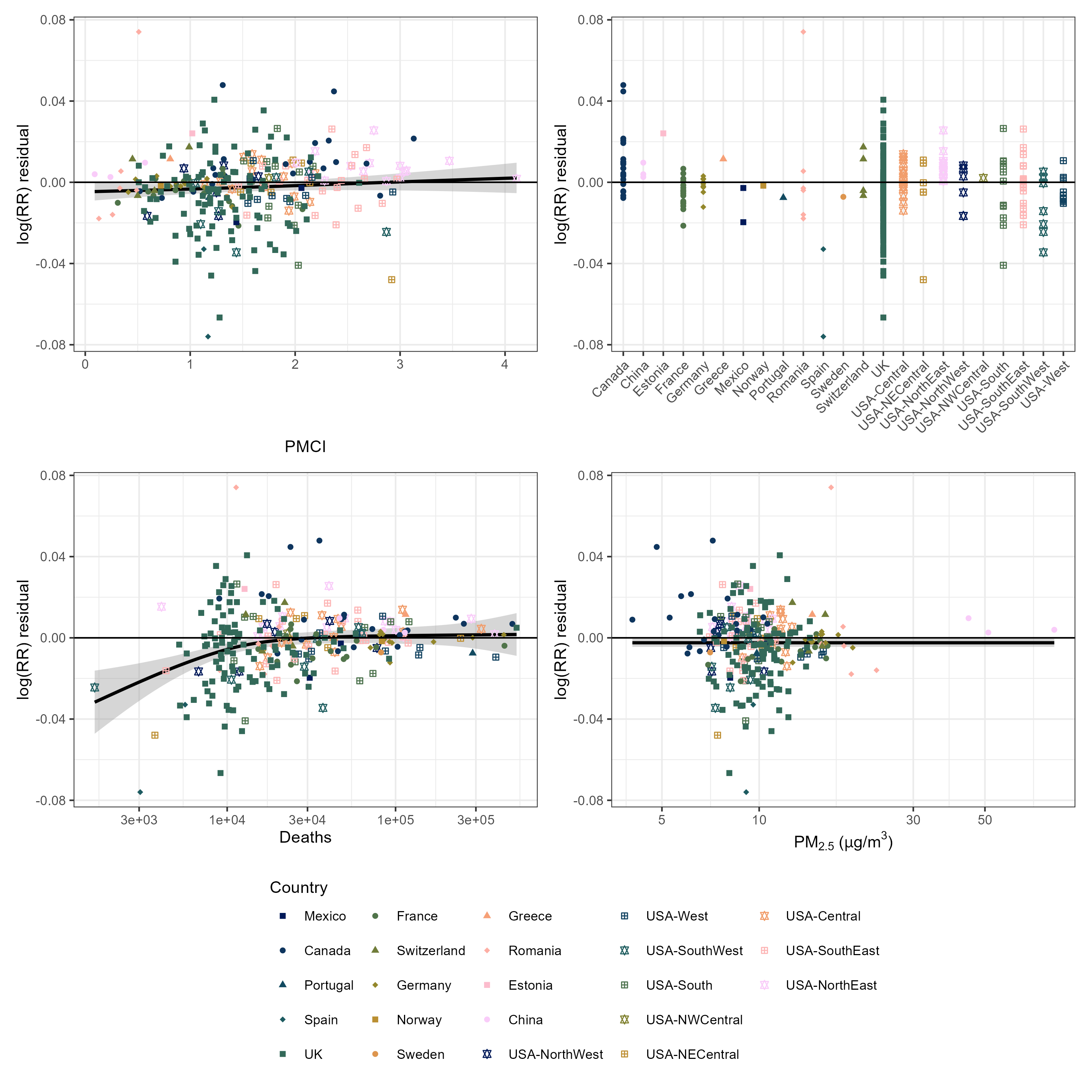


**Figure S****3.** Marginal residuals from the fitted second-stage meta-regression models versus PMCI (topleft), PM_2.5_ (topright), total deaths from the location (bottom left) and country (bottomright).

# Sensitivity Analysis

There is evidence of short-term effects of Ozone (O_3_) and nitrogen dioxides (NO_2_) on mortality independently from PM_2.5_,^4–6^ while they also tend to be correlated since reacting in the atmosphere. As a sensitivity analysis, we performed an analysis in which both O_3_ and NO_2_ are controlled for by adding their lag 0-1 moving average as a linear term in the first-stage analysis.

Figure S4 compares the resulting first-stage RR from this sensitivity analysis, to the first-stage RR from the main analysis. Overall, there is a slight attenuation with 60% of cities having lower RRs when adjusted for O_3_ and NO_2_ and an average difference of RRs of 0.0034. There are two main outliers that are Galati (Romania) and Seattle (US). Since daily O_3_ and NO_2_ data are not available everywhere, this reduces the number of cities in the analysis to 133, resulting in a slight loss of power as shown in Table S5.


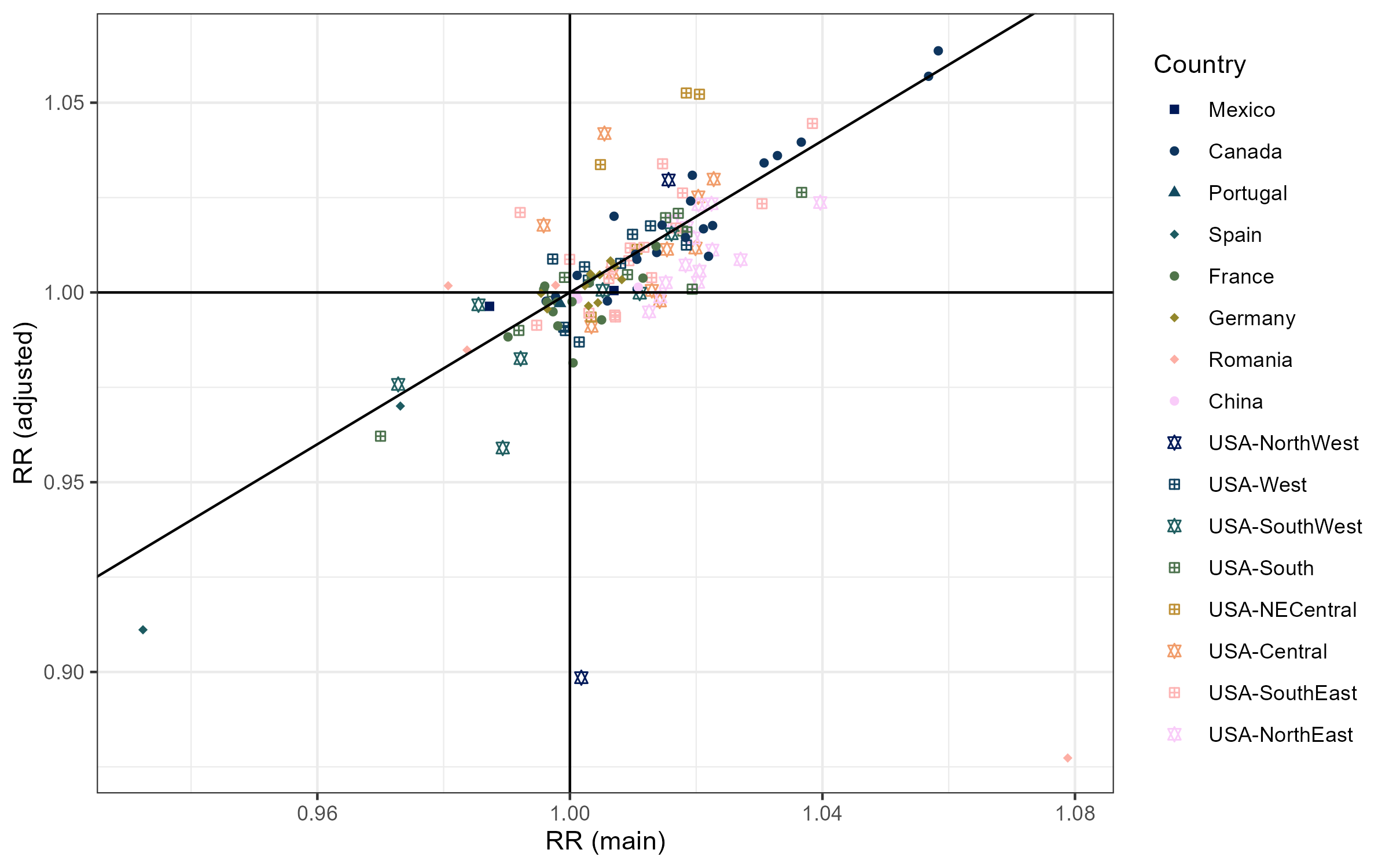


**Figure S****4.** First-stage relative risks (RR) adjusted for O3 and NO2 versus RRs used in the main analysis.

**Table S****5.** Results of the analysis with RR adjusted for both O_3_ and NO_2_. Table includes the same information as Table 2 in the main manuscript.

| Model |  | RER (95% CI) | LRT P-value | AIC | BIC |
| --- | --- | --- | --- | --- | --- |
| Main | PMCI | 1.0037 (1.0010 - 1.0065) | 0.0087 | -760.68 | **-744.00** |
| Null |  |  | 1.0000 | -755.98 | -742.00 |
| Gas Mixture | NO_2_ | 0.9994 (0.9986 - 1.0002) | 0.0017 | -763.44 | -733.83 |
|  | SO_2_ | 0.9992 (0.9974 - 1.0010) |  |  |  |
|  | O_3_ | 0.9947 (0.9913 - 0.9980) |  |  |  |
|  | HCHO | 1.0011 (0.9989 - 1.0033) |  |  |  |
|  | CO | 1.0053 (1.0023 - 1.0083) |  |  |  |
|  | NH_3_ | 1.0001 (0.9995 - 1.0006) |  |  |  |
| O_x_ | O_x_ | 0.9981 (0.9956 - 1.0007) | 0.1559 | -755.80 | -739.12 |
| PM_2.5_ Composition | SO_4_^2-^ | 0.9982 (0.9914 - 1.0050) | **0.0004** | **-766.99** | -737.38 |
|  | NH_4_^+^ | 1.0035 (0.9996 - 1.0074) |  |  |  |
|  | NO_3_^-^ | 0.9967 (0.9944 - 0.9989) |  |  |  |
|  | BC | 1.0043 (1.0018 - 1.0067) |  |  |  |
|  | OC | 1.0007 (0.9983 - 1.0032) |  |  |  |
|  | SS | 1.0002 (0.9934 - 1.0071) |  |  |  |
|  | DUST | 0.9972 (0.9819 - 1.0128) |  |  |  |

# References

1. Brook, J. R., Kharol, S. K., Shephard, M. W., Sioris, C. E. & McLinden, C. A. Characterization of Air Pollution Mixtures across the Northern Hemisphere to inform a Multi-Pollutant Index. *Rev.* (2024).

2. Raftery, A. E. Bayesian Model Selection in Social Research. *Sociol. Methodol.* **25**, 111–163 (1995).

3. Burnham, K. P. & Anderson, D. R. Multimodel Inference: Understanding AIC and BIC in Model Selection. *Sociol. Methods Res.* **33**, 261–304 (2004).

4. Orellano, P., Reynoso, J., Quaranta, N., Bardach, A. & Ciapponi, A. Short-term exposure to particulate matter (PM10 and PM2.5), nitrogen dioxide (NO2), and ozone (O3) and all-cause and cause-specific mortality: Systematic review and *meta*-analysis. *Environ. Int.* **142**, 105876 (2020).

5. Vicedo-Cabrera, A. M. *et al.* Short term association between ozone and mortality: global two stage time series study in 406 locations in 20 countries. *BMJ* **368**, (2020).

6. Meng, X. *et al.* Short term associations of ambient nitrogen dioxide with daily total, cardiovascular, and respiratory mortality: multilocation analysis in 398 cities. *BMJ* **372**, n534 (2021).
